# A laboratory study on fault slip caused by fluid injection directly versus indirectly into a fault: implications for induced seismicity in EGSs

**DOI:** 10.1098/rsta.2023.0186

**Published:** 2024-08-09

**Authors:** Supeng Zhang, Yinlin Ji, Hannes Hofmann, Shouding Li, Erik Rybacki, Günter Zimmermann, Arno Zang

**Affiliations:** ^1^ Helmholtz Centre Potsdam GFZ German Research Centre for Geosciences, Telegrafenberg, Potsdam 14473, Germany; ^2^ Key Laboratory of Shale Gas and Geoengineering, Institute of Geology and Geophysics, Chinese Academy of Sciences, Beijing 100029, People’s Republic of China; ^3^ College of Earth and Planetary Sciences, University of Chinese Academy of Sciences, Beijing 100049, People’s Republic of China; ^4^ Institute of Applied Geosciences, Technische Universität Berlin, Ernst-Reuter-Platz 1, Berlin 10587, Germany; ^5^ Institute of Geosciences, Potsdam University, Karl-Liebknecht-Str. 24-25, Potsdam 14476, Germany

**Keywords:** enhanced geothermal system, hydraulic stimulation, induced seismicity, hydro-fracturing, hydro-shearing

## Abstract

Enhanced geothermal systems (EGSs) developed by hydraulic stimulation are promising for exploiting petrothermal heat by improving fluid pathways in low-permeable geothermal reservoir rocks. However, fluid injection into the subsurface can potentially cause large seismic events by reactivating pre-existing faults, which is a significant barrier to EGSs. The management of injection-induced seismicity is, therefore, essential for the success of EGSs. During the hydraulic stimulation of an EGS, fluid can be injected into a fault zone or into the rock matrix containing pre-existing faults adjacent to the injection well. The differences in hydromechanical responses between fluid injection into and adjacent to a fault have not been investigated in detail. Here, we performed triaxial fluid injection experiments involving injecting fluid directly and indirectly into a fault in granite rock samples to analyse the distinct hydromechanical responses and estimate the injection-induced seismicity in both cases. Our results suggest that in addition to directly injecting fluid into a critically stressed fault, injecting into nearly intact granite adjacent to the fault could also cause injection-induced seismic hazards owing to the high fluid pressure required to create new fractures in the granite matrix. It is, therefore, important to carefully identify pre-existing faults within tight reservoirs to avoid injecting fluid adjacent to them. Additionally, once prior unknown faults are delineated during hydraulic stimulation, appropriate shut-in strategies should be implemented immediately to mitigate seismic risks.

This article is part of the theme issue ‘Induced seismicity in coupled subsurface systems’.

## 1. Introduction

The demand for green and renewable energy has been growing because of global warming and pollution caused by fossil fuels. Geothermal energy is considered as a highly competitive energy source that is potentially capable of meeting this demand [1,2]. Of all the types of geothermal resources, hot-dry-rock (HDR) reservoirs where the heat is stored in low-permeability rock masses (i.e. petrothermal energy), have the greatest potential and the best prospects for development because of their wide distribution and high enthalpy [3]. However, despite the high temperature [4], HDR (particularly granitic HDR) reservoirs are generally characterized by low porosity and permeability [5]. Consequently, it is challenging to exploit the heat stored in an HDR reservoir directly, and, therefore, constructing an enhanced geothermal system (EGS) by hydraulic, chemical or thermal stimulation was proposed as a solution [6,7]. To construct an EGS, hydraulic stimulation, involving the injection of fluids into HDR reservoirs, is prevalently used [8–10] and currently indispensable for increasing reservoir permeability to improve the extraction efficiency of geothermal heat [11,12]. However, it is recognized that fluid injection into the subsurface has the potential to induce seismic events by reactivating pre-existing faults, which is a significant barrier to the development of EGSs [13–15]. For instance, the hydraulic stimulations of low-permeability HDR reservoirs have induced an *M*_L_ (local magnitude) 3.4 earthquake in Basel, Switzerland [10], an *M*_L_ 2.9 earthquake in Soultz-sous-Fôrets, France [9] and an *M*_W_ (moment magnitude) 5.5 earthquake in Pohang, South Korea [14]. Hence, mitigating and preventing induced seismic hazards are pivotal to the success of an EGS.

**Figure 1.**
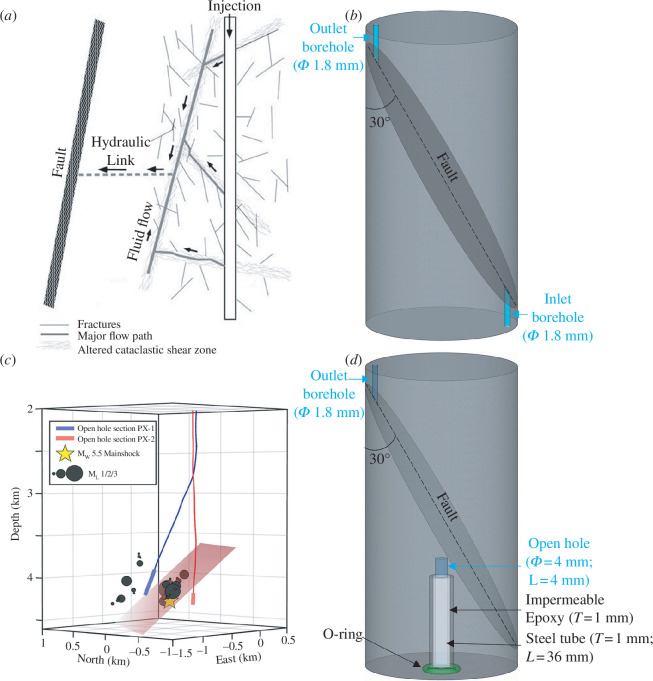
Two typical types of enhanced geothermal systems and the corresponding sample geometries simulated in the laboratory. (*a*) Schematic diagram illustrating the concept of fluid injection (nearly) directly into a pre-existing fractured zone, providing a flow path for fluid migration towards a far field fault, analogous to the geological configuration of the Soultz-sous-Forêts EGS site (adapted from Evans *et al*. [18]). (*b*) Geometry of the granite sample in the direct injection case with inlet and outlet boreholes allowing water to flow through the inclined fault. (*c*) The dimensions and location of the fault (red rectangle) on and around which induced seismic events occurred, as well as the trajectories of the two injection wells (PX-1 and PX-2) at the Pohang EGS site (adapted from Yeo *et al*. [20]). (*d*) Geometry of the granite sample in the indirect injection case containing an inclined fault, an outlet borehole and an inlet borehole consisting of a steel tube wrapped by impermeable epoxy and a section of open hole. The O-ring was used to seal the gap between the endcap and the sample.

According to whether or not new fractures are created to hydraulically connect the injection well and pre-existing faults [16,17], there are two typical *in situ* scenarios of hydraulic stimulation for an EGS. In the first scenario, fluid injected through the injection well could easily pressurize the pre-existing fault zone, leading to the reduction of the effective normal stress, and potentially induce fault reactivation and seismicity [18,19]. For example, in the EGS at Soultz-sous-Fôrets, France, the fluid injected into a fractured zone through the injection well pressurized the nearby sub-vertical fault through the hydraulic link provided by the natural flow paths between the fault and the injection well (figure 1*a*). The accumulation of fluid pressure in the sub-vertical fault ultimately led to seismic events [18,21]. Besides the potential seismic hazards, natural fault damage zones can also be targets for geothermal drilling, as they can be natural permeable channels within low-permeability geothermal reservoirs [22–24]. For instance, in the United Downs Deep Geothermal Power project, two wells were drilled into the Porthtowan fault in Cornwall, UK, to circulate fluid through this major fault zone [19,25]. In the second scenario, there was little or no hydraulic link between any pre-existing faults and the injection well prior to hydraulic stimulation, and the fluid injected into the injection well was unable to pressurize the nearby fault until new fluid pathways between the well and the fault were created [17]. Note that the latter scenario targets the low-permeability reservoir but not the fault. A typical example of this scenario is the Pohang EGS in South Korea. Particularly, the low-permeability reservoir rock between the open-hole sections of the two injection wells (PX-1 and PX-2) was intersected by an initially unidentified fault zone, which hosted the 2017 *M*_W_ 5.5 Pohang earthquake (figure 1*c*). Approximately 12 000 m^3^ of water was injected into PX-1 and PX-2 for the hydraulic stimulation, and numerous seismic events including the largest *M*_W_ 5.5 earthquake were induced during and after the fluid injection [14,20,26].

There have been plenty of experimental studies on the hydro-shearing of pre-existing faults [13,27,28], in which fluid was directly injected into a fault in a rock sample to induce fault slip. There have also been many studies on hydro-fracturing [29–31], in which fluid was injected into rock matrix to create new fractures. The combination of hydro-fracturing and hydro-shearing, in which fluid is injected into rock matrix adjacent to a fault to create hydraulic fractures and the newly created fractures propagate towards the nearby fault, has also been studied and mainly focused on the fracture propagation path (i.e. arrest/cross/offset) relative to the pre-existing fault as well as the geometry of the eventual fracture network [32–34]. A numerical study, using a particle flow code, on the interplay between hydro-fracturing and hydro-shearing by Yoon *et al*. [35] investigated not only the geometry of the fracture network but also the mechanisms of microseismic events induced by stress shadowing and hydro-shearing. The triaxial hydraulic stimulation experiments on polymethyl methacrylate samples under relatively low confining stresses by Mighani *et al*. [34] indicated that the slip of natural fractures could be triggered by the propagating hydraulic fracture, and it is hydro-shearing rather than hydro-fracturing that can cause the abrupt drop in injection pressure. Furthermore, the magnitude of seismicity induced by hydro-fracturing is negligible compared to that of hydro-shearing. Recent triaxial experimental studies focusing on the interaction of hydro-fracturing and hydro-shearing in shale and granite samples under more realistic stress conditions suggested that the stress perturbation caused by the propagating hydraulic fracture could cause the slip of a pre-existing fault even before the fracture reached the fault [32,36]. Furthermore, an experimental study focusing on the hydromechanical behaviour of pre-existing fractures demonstrated that hydro-shearing and hydro-fracturing can occur simultaneously in EGS stimulation, and that mixed-mode fracture propagation can occur at a pore pressure below the minimum principal stress, resulting in significant permeability enhancement owing to the form of the interconnected fracture network [37]. More recently, a cubic-metre scale laboratory study, involving both hydro-fracturing and fault reactivation, indicated that the fluid pathways created by hydro-fracturing play a pivotal role in controlling induced seismicity [38]. Nevertheless, the differences in the responses of stress, deformation, pore pressure, the deformation moment released by fault slip [39] and the injected volume and hydraulic energy between injecting fluid directly versus indirectly into a fault, have not yet been investigated systematically. Understanding these differences could be helpful to manage the hydraulic stimulations in EGS projects to potentially alleviate hazardous seismic activities.

To reveal the differences in the hydraulic, mechanical and energy responses to hydraulic stimulations in the two typical *in situ* EGS scenarios, i.e. fluid injection directly and indirectly into a fault (figure 1*a*,*c*), we conducted triaxial fluid injection experiments on two granite samples (figure 1*b,d*). With these experiments, we intend to provide insights and implications for managing injection-induced seismicity in EGS development.

## 2. Material and methods

### (a) Sample preparation

The fluid injection experiments were conducted on Odenwald granite sourced from Rimbach, Germany. The granite is primarily composed of quartz, feldspar and mica, with an average grain size of approximately 2 mm [40]. This granite is characterized by a porosity of approximately 0.6% and displays macroscopic homogeneity. The permeability of the granite was measured as approximately $1\times 10^{-18}m^{2}$ under a hydrostatic pressure of 2 MPa [41]. The uniaxial compressive strength of the granite is within the range of approximately 120–140 MPa [42].

**Figure 2.**
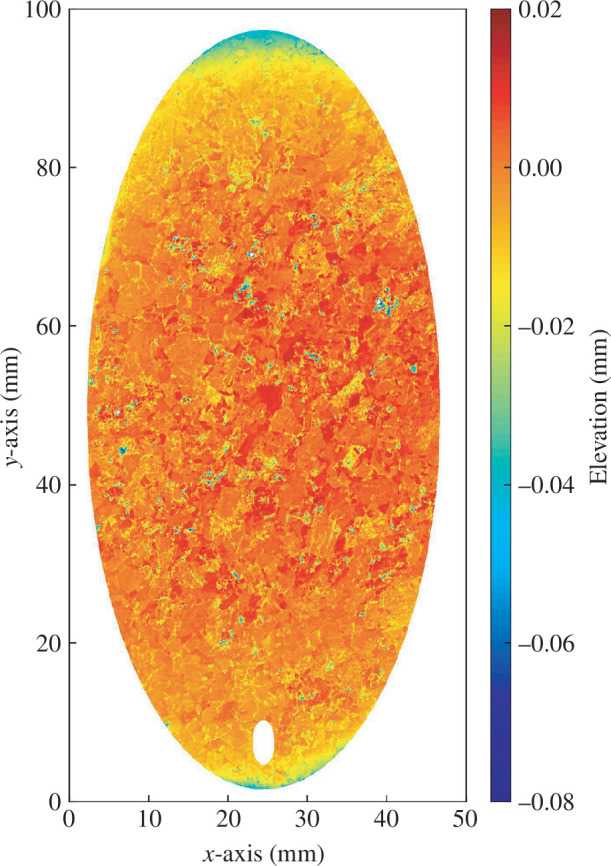
Surface morphology of the faults used in the experiments. The colour bar indicates the elevations of surface asperities. The two samples share presumably the same fault surface because they were prepared using the same method.

In this study, the samples are cylinders with a diameter of 50 mm and a length of 100 mm, each containing a sawcut fault oriented 30° to the sample axis (figure 1*b,d*). The sawcut faults in both granite samples were polished using sandpaper with the same particle size of 30.2 µm. We assume that the faults of the two samples have comparable initial surface morphology owing to the same polishing process. An optical profilometer (Keyence VR-3200 3D) with a vertical precision of 1 µm and a horizontal resolution of 47 µm was used to capture the surface morphology of the sawcut faults (figure 2). The raw surface morphology data can be accessed directly [43]. The scanning data was used to compute the surface roughness parameter *Z*_2_ as [44]:


(2.1) $$Z_{\text{2}}={\left[\frac{1}{M}\sum_{i=2}^{M}{\left(\frac{z_{\text{i-1}}-z_{\text{i}}}{y_{\text{i-1}}-y_{\text{i}}}\right)}^{2}\right]}^{\frac{1}{2}},$$

where $y_{i}$ and $z_{i}$ are the horizontal coordinate and elevation of the *i*th sampling point (similarly, $y_{i-1}$ and $z_{i-1}$ are the horizontal coordinate and elevation of the (*i*−1)th sampling point) on a two-dimensional fracture profile along the shear direction (i.e. *y* direction in figure 2), respectively; *M* is the total quantity of the sampling points along the profile. The average *Z*_2_ value of 10 evenly spaced two-dimensional profiles parallel to the shear direction was regarded as the *Z*_2_ value of the whole three-dimensional sawcut fault surface. Apart from *Z*_2_, the root mean square (RMS) asperity height of the sawcut fault, which represents the general height of the asperities distributed on the fault surface, was also evaluated. The *Z*_2_ value and RMS asperity height of the three-dimensional fault surface were calculated as 0.072 and 0.010 mm, respectively. The two ends of the granite samples were polished to be flat and parallel within ±0.1 mm.

For the sample used to conduct the direct fluid injection experiment where water was injected directly into the fault, two boreholes with a nominal diameter of 1.8 mm were drilled parallel to the sample axis, intersecting the fault surface close to the ends of the sample, to allow water to flow through the inclined fault from the inlet borehole to the outlet borehole (figure 1*b*). The rock matrix of the sample remained nearly intact and impermeable during the experiment as a result of the favourable angle of the fault as well as the high strength and low permeability of the granite matrix; fluid flow remained confined within the fault plane [41].

To prepare the granite sample for the indirect fluid injection experiment in which water was injected into the rock matrix adjacent to the fault, a borehole with a nominal diameter of 1.8 mm (outlet borehole) was drilled parallel to the sample axis into the upper sample half, connecting the fault surface to facilitate the water communication between the upper endcap and the fault surface (figure 1*d*). In addition, an inlet borehole, lined with a steel tube of thickness 1 mm and of length of 36 mm, wrapped in a 1 mm thick layer of impermeable epoxy, was inserted. An open-hole section of diameter of 4 mm and length 4 mm was drilled from the end surface centre of the lower sample half to inject water into the sample (figure 1*d*). The distance between the end of the open hole and the fault was approximately 5.7 mm. The rock matrix between the end of the open hole and the fault was initially intact and expected to be hydraulically fractured by the injected water during the fluid injection experiment [32,34,38].

### (b) Experimental methods

The samples and steel endcaps were encased within a neoprene jacket and subsequently placed in the triaxial cell of the MTS 815 rock mechanics test system. To rule out the effect of the buildup of air pressure (rather than water pressure) on our experimental results, we expelled the air from the samples prior to the experiments by vacuumizing and saturating them sequentially. First, the samples were exposed to a vacuum of 1 kPa for one day. Subsequently, they were fully saturated by injecting distilled water into both injection and monitoring boreholes until the injected volume did not increase at a fluid pressure of 0.2 MPa and hydrostatic confining pressure of 2 MPa, which typically lasted overnight. Prior to commencing the experiment, the samples were subjected to a preparatory process involving the cyclic alteration of the confining pressure within the range of 2–26 MPa to eliminate the misalignment and irreversible plastic deformation of the samples [45].

On each sample, we conducted a triaxial fluid injection experiment. During each experiment, an initial triaxial shearing stage was performed at a constant axial displacement rate of 1 μm s^−1^ to determine the peak differential stress ( $\sigma_{d}^{p}$ ) of the sample. This test was performed under a constant confining pressure of 21 MPa and a uniform pore pressure of 1 MPa. The differential stress was then reduced to 90% of the peak differential stress by slowly retracting the axial piston. Subsequently, a fluid injection stage was performed under a stress relaxation condition.

The stress relaxation condition was first used by Rutter *et al*. [46] and Rutter & Mainprice [47] to study the rheology of geological materials. In their experiments, the axial piston was adjusted to compensate for the sample length change caused by the testing machine relaxation, thereby holding the sample length constant. However, these experiments were actually performed under an artificially controlled stress path and, as such, the boundary condition cannot characterize the stress relaxation during fault reactivation in the upper crust [37]. To address this issue, Ye and Ghassem [28] and Ye *et al*. [48] proposed a constant piston displacement control method, which was also used in this study, to investigate the hydromechanical responses of the pre-existing fault subject to fluid injection.

During the fluid injection stage under the stress relaxation condition with a constant axial piston position, the axial piston was fixed and distilled water was injected through the inlet borehole at a rate of 0.2 ml min^−1^, with concurrent monitoring of the pore pressure close to the outlet borehole. The elevation of pore pressure induced the slip of the pre-stressed sawcut fault (as well as hydraulic fracture in rock matrix in the indirect injection case) and led to axial stress relaxation. The fluid injection was stopped upon the observation of a full slip event after which the differential stress on the sample stabilized.

The axial force was measured using an in-vessel load cell with an accuracy of ±0.3%. The total axial displacement was monitored using a linear variable displacement transducer located outside the pressure vessel with an accuracy of ±0.5%. The fluid pressures within the inlet and outlet boreholes (i.e. the injection and monitoring pressures) were recorded with two pressure sensors integrated within the (Quizix Q6000-20K) pump. The value of sample shortening ( $L_{s}$ ) can be calculated from the total axial displacement ( D ), the axial force ( F ) and the vertical stiffness of the testing system ( $k_{sys}$ ≈ 796 kN mm^−1^) using [41]


(2.2) $$L_{\text{s}}=D-\frac{F}{k_{\text{sys}}}.$$

The data pertaining to stress, deformation and hydraulic parameters were recorded at a sampling rate of 10 Hz throughout the experiments. The sample shortening rate was calculated as the first derivative of the sample shortening with respect to time at an interval of 0.1 s. After the experiment on the sample where fluid was injected indirectly into the fault, we analysed the sample using an X-ray CT scanner (GE Phoenix Nanotom 180 NF) to characterize the hydraulic fracture induced during the fluid injection stage. The experimental data in this study are directly available at [43].

## 3. Results

### (a) Hydromechanical responses of fluid injection directly into a fault

**Figure 3.**
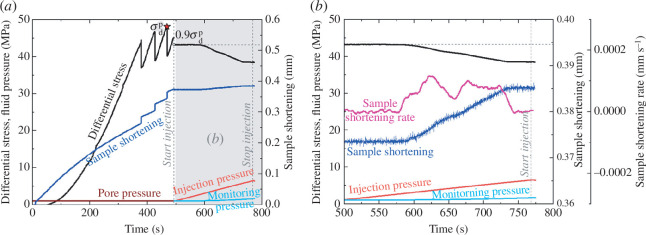
Experimental results of fluid injection directly into a fault in a granite sample. (*a*) Time history of differential stress, sample shortening and pore pressure (including injection and monitoring pressures) during the triaxial fluid injection experiment on the granite sample associated with injecting distilled water directly into a fault. The experiment consists of a triaxial shearing stage and a subsequent fluid injection stage starting at 493 s; $\sigma_{d}^{p}$ indicates the peak differential stress obtained in the triaxial shearing stage. The light grey area indicates the time range shown in (*b*). (*b*) Zoomed-in view of the injection-induced slip event in (*a*). Apart from the parameters depicted in (*a*), the sample shortening rate is also presented to better elucidate the slip characteristics. The sample shortening rate was smoothed using Savitzky–Golay filter to remove high-frequency noise.

The mechanical and hydraulic results of the triaxial fluid injection experiment involving injecting fluid directly into a fault are presented in figure 3. The experiment includes a triaxial shearing stage and a subsequent fluid injection stage. In the triaxial shearing stage, three unstable stick-slip events were observed with abrupt differential stress (the difference between axial stress and confining pressure) drops of approximately 7.6, 7.9 and 8.0 MPa (figure 3*a*). In addition, sudden sample shortening jumps of approximately 0.02, 0.02 and 0.03 mm were observed when the stick-slip events occurred. The pore pressure (including both injection and monitoring pressures) on the fault was uniform at 1 MPa during the entire triaxial shearing stage. Considering shear hardening associated with increasing slip displacement [41,49], the peak differential stress ( $\sigma_{d}^{p}$ ) of the sample was determined as 48.14 MPa at the last stick slip.

Afterwards, the differential stress was reduced to 90% of the peak differential stress measured in the triaxial shearing stage ( $0.9\sigma_{d}^{p}$ in figure 3*a*). The subsequent fluid injection stage, starting at 493 s from the beginning of the experiment, was performed under the stress relaxation condition with a constant axial piston position (cf. the studies by Ye *et al*. [48], Ye & Ghassemi [28] and Ji *et al*. [41]). In the fluid injection stage, we observed an injection-induced stable sliding of the fault accompanied by a gradual reduction in differential stress of approximately 4.7 MPa and a gradual increase in the sample shortening of approximately 0.01 mm (figure 3*b*). The peak sample shortening rate of this stable sliding event was approximately 1.17 × 10^−4^ mm s^−1^, which is extremely low when compared with the sample shortening rate of stick-slip events [41]. The monitoring pressure gradually and slightly increased with the continuously increasing injection pressure. The observed lag in the increase of monitoring pressure relative to the injection pressure indicates a heterogeneous fluid pressure distribution across the fault surface [41,50,51].

### (b) Hydromechanical responses of fluid injection indirectly into a fault

**Figure 4.**
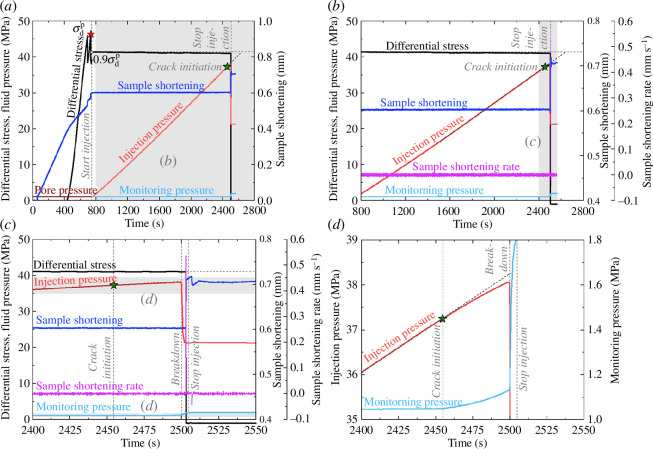
Experimental results of fluid injection indirectly into a fault in a granite sample. (*a*) Time history of the differential stress, sample shortening and pore pressure (including injection and monitoring pressures) during the triaxial fluid injection experiment on the granite sample associated with injecting distilled water indirectly into a fault. The experiment consists of a triaxial shearing stage and a subsequent fluid injection stage starting at 745 s. The $\sigma_{d}^{p}$ depicted by the red star indicates the peak differential stress obtained in the triaxial shearing stage. The light grey area indicates the time range shown in (*b*). (*b*) Zoomed-in view of the fluid injection stage in (*a*). In addition to the parameters presented in (*a*), the sample shortening rate is also shown to better elucidate the deformation characteristics. The light grey area presents the time range shown in (*c*). (*c*) Zoomed-in view of the injection-induced crack initiation and breakdown events during the fluid injection stage in both (*a*) and (*b*). The light grey areas depict the pressure ranges shown in (*d*). (*d*) Enlarged temporal evolution of injection and monitoring pressures within the time scale specified in (*c*). The linear trend of injection pressure is indicated by the dark grey dashed line. The time of crack initiation, breakdown and stopping injection is denoted by the light grey dashed lines and the text close to them. The four green stars depict crack initiation indicated by the onset of injection-pressure deviation from the linear trend.

Figure 4 shows the hydraulic and mechanical results of the triaxial experiment where fluid was injected indirectly into the fault in the granite sample. This experiment also consists of a triaxial shearing stage and a subsequent fluid injection stage, which is the same as the direct injection case. During the triaxial shearing stage, two unstable stick-slip events were observed with sudden differential stress drops of approximately 7.6 and 8.3 MPa (figure 4*a*). Furthermore, abrupt increments in sample shortening of approximately 0.03 mm each were also recorded concomitantly with the onset of the two stick-slip events. The pore pressure, including both injection and monitoring pressures, on the fault was held constant and uniform at 1 MPa throughout the entire triaxial shearing stage. The peak differential stress ( $\sigma_{d}^{p}$ ) of the sample was measured as 46.37 MPa at the end of the shearing.

**Figure 5.**
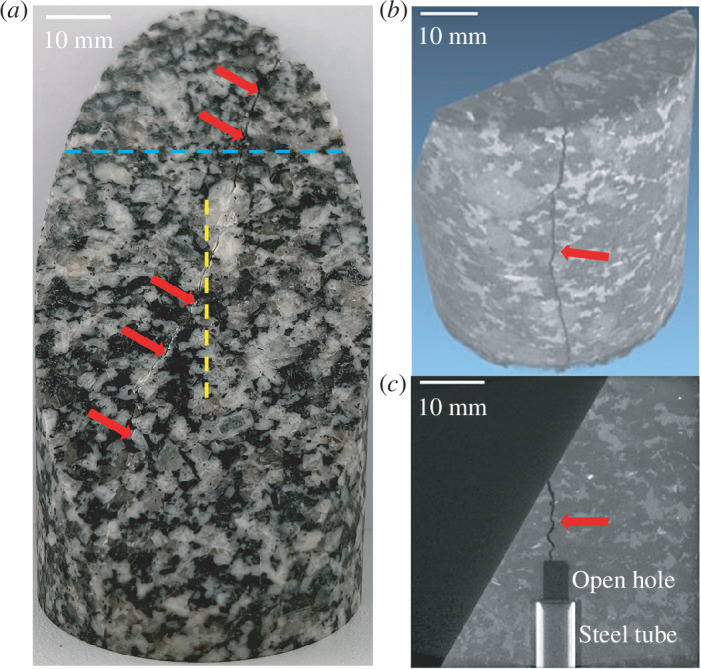
The granite sample after the fluid injection experiment where fluid was injected indirectly into a fault. (*a*) Fault surface of the lower sample half after the experiment. The blue dashed line indicates the upper bound of the CT image in (*b*). The yellow dashed line represents the position of the section in (*c*). The red arrows indicate the fracture induced by fluid injection during the experiment. (*b*) The three-dimensional digitally reconstructed sample based on the CT images. (*c*) The CT image of a cross-section of the lower sample half.

Following the triaxial shearing, we reduced the differential stress to 90% of the peak differential stress ( $0.9\sigma_{d}^{p}$ in figure 4*a*). The subsequent fluid injection stage was performed after 745 s relative to the start of the whole experiment under the stress relaxation condition with the axial piston fixed, which is similar to the direct injection case. In the fluid injection stage, an injection-induced fracture (hydraulic fracture) initiated from the end of the open-hole section of the injection hole (figure 5*c*) at 2455 s from the beginning of the experiment, which is signified by the deviation of the injection pressure from a linear trend (figure 4*d*). The hydraulic fracture propagated near-vertically to the pre-existing fault, and the fracture length between the end of the open hole and the fault was approximately 10 mm (figure 5*c*). It is notable that the hydraulic link between the fracture and the fault was created almost immediately once the fracture initiated because of the small distance between the open hole section and the fault, which is signified by the increasing monitoring pressure after crack initiation (figure 4*d*). Nevertheless, after crack initiation, the hydraulic fracture was still able to propagate three-dimensionally in the granite matrix until sample breakdown, manifested by the abrupt drop in injection pressure from approximately 38 to 21 MPa (equal to the confining pressure) within approximately 3 s (figure 4*b*,*c*), during which the intersection length between the fault and fracture was extended. When the hydraulic fracture was propagating, the monitoring pressure gradually and slightly increased from 1.05 to 1.13 MPa (figure 4*d*), meaning that the newly created hydraulic link between the injection hole and the inclined fault was improved. At the time of breakdown, signified by a sudden drop of injection pressure (figure 4), the hydraulic fracture extended to not only the fault surface but also the outer surface of the sample (figure 5*a*,*b*) producing a visible macroscopic fracture that resulted in the injection pressure falling to the confining pressure and a sudden rise of the monitoring pressure from 1.13 to 1.74 MPa (figure 4*c*). The monitoring pressure continued increasing slowly even after the breakdown until the stop of injection (figure 4*c*). Additionally, 3.2 s after the onset of breakdown, we observed a stick-slip event caused by the dramatically increased fluid pressure at the fault, accompanied by an abrupt drop in the differential stress of approximately 41 MPa and an abrupt rise in the sample shortening of approximately 0.1 mm at approximately 2503 s (figure 4*c*). The peak sample shortening rate of this stick-slip event was approximately 0.54 mm s^−1^ (figure 4*b*,*c*), which is approximately three orders of magnitude higher than the peak sample shortening rate of the stable sliding event in the direct injection case. Although the peak sample shortening rates of the slip events may be underestimated owing to the low recording rate of 10 Hz [52,53], our dataset supports the relative comparison in the magnitude of the peak rates. It is worth noting that the reduction of the differential stress is so significant that the differential stress on the sample decreased to zero after the slip event, meaning that the sample was fully relaxed (figure 4*b*,*c*). Finally, the injection was immediately stopped 1.7 s after the occurrence of the slip event (figure 4*c*).

As shown in figure 4*d*, prior to crack initiation, the monitoring pressure of 1.05 MPa was slightly higher than the initial value of 1 MPa with a linear increase of the injection pressure even though there was no fracture between the injection hole and the inclined fault. This subtle increase in the monitoring pressure might have been caused by fluid diffusion within the granite matrix, fluid compression in the fault owing to the pressure accumulation in the injection hole or both [38,54,55]. In addition, the injection pressure and the confining pressure are identical after breakdown as a result of the connection between the injected fluid and confining oil caused by the macroscopic fracture (figures 4*c* and 5). However, the monitoring pressure after breakdown could not reach the value of the confining pressure (figure 4*a–c*), which is probably because the macroscopic hydraulic fracture did not connect to the outlet borehole in the sample (figure 5*a*).

## 4. Discussion

### (a) Differences in the hydromechanical responses of the two samples

There are some significant differences in the hydromechanical responses to fluid injection directly and indirectly into a fault under pre-stressed state in the granite samples. With the direct fluid injection, the monitoring pressure slowly but continuously increased during injection because the injection and monitoring holes were hydraulically connected by the sawcut fault during the whole experiment (figure 3*b*). In contrast, during indirect fluid injection, the monitoring pressure evolution can be divided into four stages, including the almost constant stage before crack initiation, the gradual increase stage between crack initiation and breakdown, the abrupt rise stage between breakdown and injection termination and the constant stage after stopping injection (figure 4*b–d*).

When the inclined sawcut fault was reactivated in the direct injection case, the differential stress on the sample was slightly reduced by approximately 4.7 MPa during a stable sliding event (figure 3*b*). The stable sliding might be the result of the low effective normal stress and low critical rheological stiffness of the fault [41,56]. In the case where fluid was injected indirectly into the fault, the differential stress dropped dramatically by approximately 41 MPa to zero (figure 4*b,c*), meaning that the differential stress in this case was fully released by a stick-slip event. Although fluid injection directly into the fault induced a stable sliding event in this study, it is also possible that stick-slip events could be induced in other direct injection cases, depending on the stiffness contrast between the sample and the loading frame, as well as the friction rate parameters of the fault [13]. However, regardless of whether stable sliding or stick slip was induced in the direct injection case, residual differential stress can persist after the slip event, i.e. the differential stress on the sample cannot be immediately reduced to zero by the event [28,41]. The peak sample shortening rate of the stick-slip event in the indirect injection case (figure 4*c*) was approximately three orders of magnitudes higher than that of the stable sliding event in the direct injection case (figure 3*b*) and of the stick-slip events under similar experimental conditions of direct injection [41]. Furthermore, the sample shortening during the stick-slip event in the indirect injection case (figure 4*b*,*c*) exceeded that of both stable sliding (figure 3*b*) and stick-slip [41] events in direct injection cases by approximately 10 orders of magnitude. The time delay of 3.2 s between the slip event and breakdown in the indirect injection case (figure 4*c*) indicates that the slip occurred after breakdown, suggesting that the fluid pathway provided by the hydraulic fracture is necessary for the fast slip event and strong pressure drop. This is consistent with the observation of the cubic-meter scale laboratory study by Oye *et al*. [38]. The mechanical responses of fluid injection into granite samples suggest that the fault reactivation, in the case where fluid was injected indirectly into a fault and a hydraulic fracture was created to connect the injection well and fault, is associated with larger stick-slip stress drops and higher sample shortening rates, compared to injecting fluid directly into a fault. However, there are many factors that may influence the violence of fault slip in the indirect injection case, including, for example, fault roughness, injection rate, distance between the injection well and fault, breakdown pressure of the rock matrix, as well as the aperture of the newly formed tensile fracture. Particularly, in the indirect injection case, once the hydraulic fracture connected the sawcut fault and the injection hole, the fluid pressure on the fault could be heterogeneously raised in a very short period to much higher pressures than the pressure required to reactivate the fault under a pre-stressed state. This is because of the overpressure in the injection hole required to initiate hydro-fracturing [13,50,51]. As a result, the fault was violently reactivated in this case and the differential stress was completely released (figure 4*b*,*c*). In EGS stimulation, hydro-shearing and hydro-fracturing can occur collectively in reservoir rocks, and the mixed-mode fractures can propagate at pore pressures below minimum principal stresses [37]. Nevertheless, the mixed-mode propagation pressure can be higher than the pressure required by the initiation of fault slip in the direct injection case (cf. Ye and Ghassemi [37] and Ji *et al*. [41]). Therefore, the mixed-mode fracture propagation in indirect injection cases might also induce relatively more violent reactivation of pre-existing faults. If the permeability of the rock matrix were higher, the fluid viscosity lower, or the distance between the open hole and the fault shorter than those in our experiment, the fault in the indirect injection case might also be reactivated by fluid pressure diffusion from the injection hole to the fault without overpressure in the injection hole or any hydraulic fracture, which has been already observed in prior experiments [38,54]. Therefore, the fault slip in such an indirect injection sample without overpressure would be safer than the stick slip in the indirect injection case with overpressure in this study. In summary, in addition to injecting fluid directly into a fault, injecting indirectly/adjacent to a fault can also induce seismicity and be dangerous.

### (b) Energy budget in fluid injection

**Figure 6.**
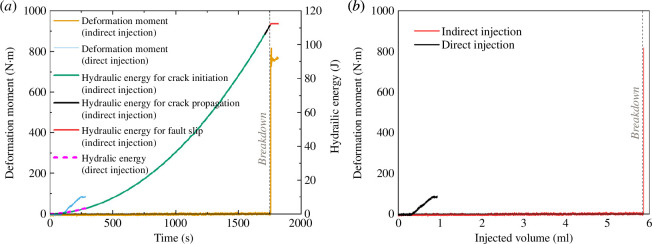
(*a*) Temporal evolution of deformation moment and hydraulic energy during the fluid injection stage of the triaxial fluid injection experiments involving injecting distilled water directly and indirectly into a fault. The evolution of hydraulic energy of the fluid injection stage in the indirect injection case was divided into three segments, including the hydraulic energy for crack initiation, the hydraulic energy for crack propagation and the hydraulic energy for sample breakdown (fault reactivation). The hydraulic energy of the fluid injection stage in the direct injection case was all used to reactivate the pre-existing fault. Time zero in (*a*) indicates the start of injection (i.e. 493 s in the direct injection case and 745 s in the indirect injection case). The gray dashed line indicates the time for breakdown of the granite sample in the indirect injection case. (*b*) The relationship between the deformation moment and injected volume during the fluid injection stage of the fluid injection experiments involving injecting distilled water directly and indirectly into a fault. The gray dashed line denotes the injected volume at which the granite sample in the indirect injection case broke down.


The energy budget can potentially be used to evaluate the deformation moment released by injection-induced fault slip, and can be estimated by examining the interplay between deformation moment and hydraulic energy [39,57,58]. The deformation moment [39] in this study is considered released mainly from fault slip, characterized by the reduction of differential stress and the increase of sample shortening, during fluid injection (figures 3*b* and 4*b,c*). To evaluate the deformation moment released by fault slip, we first need to assess the fault slip displacement [41]. Owing to the time lag of 3.2 s between the abrupt drop in the differential stress and the onset of breakdown in the indirect injection case (figure 4*c*), we reasonably presume that the drop in the differential stress and the jump in the sample shortening were mainly caused by the fault slip rather than crack initiation or propagation. The average fault slip displacements (*u*) of the direct and indirect injection cases are calculated using [41]


(4.1) $$u=\left(L_{s}-\frac{FL}{EA}\right)/cos\theta ,$$

where $L_{s}$ is the sample shortening computed with equation (2.2); *E* is the Young’s modulus of the granite matrix taken as 64 GPa [41]; *L* and *A* denote the length and cross-sectional area of the cylindrical sample, respectively; θ is the fault inclination angle with respect to sample axis and is taken as 30°. Based on the fault slip displacement, we can estimate the deformation moment ( $M_{0}$ ) of the slip event as [39,59]


(4.2) $$M_{0}=G\int_{\;\;A}u_{\text{i}}dA_{\text{i}}\approx GAu,$$

where *G* is the combined rigidity of the testing system and the fault (approx. 1 GPa [60]); $u_{i}$ and $A_{i}$ are the slip displacement and area of the *i*th patch on the fault surface, respectively; *A* is the total fault surface area ( $3.9\times 10^{-3}m^{2}$ ). Since the fault area undergoing slip increases with slip front propagation, the slip on a fault is heterogeneous and develops progressively [61], and to determine the $u_{i}$ and $A_{i}$ of each patch is unrealistic. Therefore, we estimate the deformation moment as the product of the combined rigidity, total fault area and average fault slip displacement. Based on this estimation, a relative comparison of the deformation moments released in the two injection cases is feasible owing to their significant difference (figure 6). Apart from deformation moment, the hydraulic energy ( $E_{h}$ ) during injection can be calculated as [62]

(4.3) $$E_{\text{h}}=\int_{t_{1}}^{t_{2}}Pqdt,$$

where *P* and *q* represent the injection pressure and injection rate at time *t*, respectively.

Figure 6*a* shows that the final deformation moment of the injection-induced stable sliding event in the direct injection case is much lower than that of the injection-induced stick-slip event in the indirect injection case. Therefore, in our study, the injection-induced fault reactivation in the indirect injection case is much more hazardous than that in the direct injection case. All the injected fluid was used to reactivate the fault in the direct injection case (figure 6*a,b*), but in the indirect injection case, the fault was reactivated mainly by the fluid injected in the last segment of hydraulic energy (the hydraulic energy for fault slip in figure 6*a*). Furthermore, in the indirect injection case, most of the injected water was used to generate the hydraulic fracture between the fault and the injection hole, as evidenced by both the hydraulic energy for fault slip being much smaller than the hydraulic energy for crack initiation and propagation (figure 6*a*) and the injected volume before breakdown being much larger than that after breakdown and before the end of deformation release (figure 6*b*). The hydraulic energy at the time of breakdown in this case is much higher than even the highest hydraulic energy in the direct injection case (figure 6*a*). This is the result of the higher injection pressure required to create a hydraulic fracture compared to the injection pressure required to reactivate the fault in the direct injection case (figures 3*b* and 4*b*).

### (c) Implications for injection-induced seismicity in an EGS

The hydromechanical responses and energy evolution of fluid injection into the two cylindrical granite samples suggest that in addition to injecting fluid directly into a fault, injecting fluid into nearly intact rock adjacent to a critically stressed fault, accompanied by newly created fluid pathway that intersects the fault, could also induce seismic hazard in EGSs (figures 3b, 4c and 6). Therefore, our laboratory experiments suggest to clearly delineate pre-existing faults in EGS target reservoirs to mitigate seismic hazards by avoiding drilling in the near vicinity of pre-stressed large faults within the reach of hydraulic fractures (such as Pohang).

Careful geological and geophysical surveys, especially the surveys augmented by conventional statistical methods [63] or novel artificial intelligence techniques involving machine learning [64] and deep learning [65], can be used to delineate the strikes, dips, locations and scales of pre-existing faults before EGS development to avoid drilling into intact rock close to the faults. However, earthquakes often occur on hidden faults that could be detected only after injection-induced seismicity is recorded [66,67]. Installing additional seismic stations in the vicinity of EGS sites can facilitate the ongoing detection of sufficient micro-seismic clusters in both spatial and temporal dimensions [68], which could, during hydraulic stimulation, enhance the precision of identifying reactivated faults corresponding to the seismic events with low magnitudes prior to highly hazardous earthquakes [69]. Once highly hazardous faults have been detected during a hydraulic stimulation, the injected fluid in the wells close to the faults should be immediately bled off to mitigate potential injection-induced seismic hazards [70].

### (d) Limitations of this study

The hydromechanical responses of a fault subject to fluid injection may also be significantly influenced by its surface roughness. Fluid injection into a smooth sawcut fault can induce dynamic unstable slip accompanied by an abrupt stress drop at a high slip velocity. Under otherwise similar conditions, for a rough natural fault, fluid injection tends to induce stable sliding, characterized by a gradual stress decay at a low slip velocity [71,72]. To rule out the effect of fault roughness, we used laboratory-scale sawcut faults in the two granite samples prepared with the same cutting and polishing process to mimic pre-existing faults with comparable surface roughness in EGS reservoirs. Although interesting results have been obtained from this study, considering the significant influence of surface roughness on injection-induced fault behaviours, it is necessary to study the roughness-dependent hydromechanical responses of faults subject to direct and indirect injection in the future.

We injected distilled water into both samples at the same low rate of 0.2 ml/min. However, the seismo-hydro-mechanical responses of a fault to fluid injection is highly dependent on the injection rate. For instance, when the injection rate is increased, the fluid pressurization becomes localized, the nucleation lengths of laboratory-scale injection-induced earthquakes decrease, resulting in a higher slip rate and a larger slip displacement, which signifies an intensified seismic hazard [61]. In the indirect injection case, upon the intersection of the fault and newly created fracture, the high fluid pressure accumulated around the fracture tip acted locally on the fault followed by diffusion and caused the fast fault slip and a higher seismic hazard, which is similar to a high-rate injection case under a direct injection condition.

The destructive stick slip in the indirect injection case was induced by the high fluid pressure necessary for hydro-fracturing transferred to the pre-existing fault. However, this transfer could be affected by various factors in large-scale EGS reservoirs. For example, the injection well could be far from pre-existing faults, such as in the Berlín Hot Fractured Rock, El Salvador [73]. Under this condition, the hydraulic link between the injection well and the faults would be poor if the newly generated fractures are sufficiently long. As a result, it would be difficult for fluid pressure to be transferred to the faults from the injection well through the newly created fluid pathways, and the pressure buildup on the faults may not be high enough to induce hazardous stick slip. In addition, the seismic behaviour of pre-existing faults is significantly affected by stress state. If the faults are not under critical stress, the pressure threshold over which seismicity could be triggered would increase [74,75]. Therefore, the pressure buildup caused by the intersection between the newly created fluid pathways and the faults might not exceed the threshold, and destructive seismic hazards may not be induced. That is, although the indirect injection case released more shear stress and deformation moment than the direct injection case in this study, it is possible that an indirect injection scenario could pose a lower seismic risk on a pre-stressed fault. This is particularly true when the injection well is far from the fault and the fault is not critically stressed. Consequently, it is worthwhile to investigate the effect of well-fault distance and stress state on the seismo-hydro-mechanical responses of faults under both injection conditions.

## 5. Conclusion

In this study, we conducted triaxial fluid injection experiments in granite samples with fluid injection directly and indirectly into a fault to compare the hydraulic, mechanical and energy responses in these two experiments. Our results indicate that in addition to directly injecting fluid into a pre-stressed fault, indirect injection into the fault could also be hazardous. When fluid is injected into nearly intact reservoir rock adjacent to a critically stressed fault, most of the injected hydraulic energy and volume would be consumed by creating new fluid pathways/fractures rather than reactivating the fault. Fluid injection into such a sample could induce significant fault slip owing to a sudden local pressure increase on the fault caused by the intersection of the fault and newly created fractures. Therefore, to alleviate seismic hazard, it is important to carefully detect pre-existing faults in EGS sites before drilling to avoid creating fractures that can intersect a fault. If faults are detected during, rather than before hydraulic stimulation, the injection in the well close to the faults should be immediately halted and bleed-off should be carried out to mitigate seismic hazards.

## Data Availability

The dataset supporting this study can be accessed online [43].
